# Supplementary material for: Hot spot prediction in protein-protein interactions by an ensemble system
Source: BMC Syst Biol. 2018 Dec 31;12(Suppl 9):132. doi: 10.1186/s12918-018-0665-8 (PMC6311905; doi:10.1186/s12918-018-0665-8)
Supplement: Supplementary file 2 — All relevant MATLAB code. A simple Matlab implement of our predictor. (ZIP 19,742 kb) [file 12918_2018_665_MOESM2_ESM.zip › Additional file 2/Read ASA/ASA website/dbMPIKT/4U2XA.html]

 NetSurfP - Protein Surface Accessibility and Secondary Structure Predictions

  

|  |  |  |
| --- | --- | --- |
|  |  | NetSurfP - Protein Surface Accessibility and Secondary Structure Predictions  Technical University of Denmark |

  


---

```
# For publication of results, please cite:
# A generic method for assignment of reliability scores applied to solvent accessibility predictions.
# Bent Petersen, Thomas Nordahl Petersen, Pernille Andersen, Morten Nielsen and Claus Lundegaard
# BMC Structural Biology 2009, 9:51 doi:10.1186/1472-6807-9-51
#
# Column 1: Class assignment - B for buried or E for Exposed - Threshold: 25% exposure, but not based on RSA
# Column 2: Amino acid
# Column 3: Sequence name
# Column 4: Amino acid number
# Column 5: Relative Surface Accessibility - RSA
# Column 6: Absolute Surface Accessibility
# Column 7: Z-fit score for RSA prediction
# Column 8: Probability for Alpha-Helix
# Column 9: Probability for Beta-strand
# Column 10: Probability for Coil
E D  4U2XA                  1    0.927 133.537   0.845   0.003   0.003   0.994
E L  4U2XA                  2    0.403  73.771  -0.937   0.113   0.043   0.844
E E  4U2XA                  3    0.612 106.951  -0.303   0.191   0.086   0.723
E K  4U2XA                  4    0.502 103.241   0.729   0.191   0.086   0.723
E G  4U2XA                  5    0.325  25.538  -1.023   0.199   0.152   0.649
B V  4U2XA                  6    0.224  34.475   0.623   0.373   0.401   0.226
B V  4U2XA                  7    0.186  28.650   0.331   0.268   0.505   0.227
B L  4U2XA                  8    0.102  18.621   0.337   0.478   0.309   0.214
B S  4U2XA                  9    0.312  36.578  -0.283   0.649   0.163   0.188
E D  4U2XA                 10    0.462  66.632  -0.747   0.605   0.105   0.290
B L  4U2XA                 11    0.162  29.717  -0.319   0.561   0.047   0.393
B C  4U2XA                 12    0.075  10.600  -0.300   0.455   0.046   0.498
E N  4U2XA                 13    0.457  66.905   0.675   0.386   0.097   0.517
B F  4U2XA                 14    0.167  33.457  -0.090   0.428   0.171   0.402
B L  4U2XA                 15    0.142  25.982  -0.172   0.453   0.248   0.299
E V  4U2XA                 16    0.396  60.942  -0.149   0.321   0.252   0.427
E S  4U2XA                 17    0.463  54.322  -0.264   0.125   0.227   0.648
E Q  4U2XA                 18    0.497  88.693  -0.282   0.056   0.142   0.802
E T  4U2XA                 19    0.435  60.334  -0.460   0.064   0.216   0.721
B I  4U2XA                 20    0.138  25.456  -1.181   0.021   0.279   0.699
E Q  4U2XA                 21    0.511  91.211  -0.908   0.020   0.205   0.775
E G  4U2XA                 22    0.395  31.063  -1.421   0.005   0.336   0.660
B W  4U2XA                 23    0.141  34.007   0.420   0.002   0.816   0.182
E K  4U2XA                 24    0.406  83.596   0.720   0.001   0.959   0.040
B V  4U2XA                 25    0.081  12.419   0.419   0.001   0.959   0.040
E Y  4U2XA                 26    0.289  61.717   0.654   0.001   0.959   0.040
B W  4U2XA                 27    0.179  43.146  -0.920   0.001   0.900   0.099
B A  4U2XA                 28    0.221  24.354  -1.006   0.004   0.616   0.381
E G  4U2XA                 29    0.388  30.504  -1.142   0.004   0.514   0.481
B I  4U2XA                 30    0.099  18.407   0.398   0.001   0.900   0.099
E E  4U2XA                 31    0.278  48.654  -0.018   0.001   0.959   0.040
B F  4U2XA                 32    0.082  16.498   0.281   0.001   0.900   0.099
E D  4U2XA                 33    0.328  47.294  -0.246   0.003   0.718   0.279
B V  4U2XA                 34    0.062   9.514   0.628   0.004   0.420   0.576
E T  4U2XA                 35    0.337  46.700   0.143   0.020   0.205   0.775
E H  4U2XA                 36    0.372  67.649  -0.969   0.052   0.084   0.864
E K  4U2XA                 37    0.433  89.027   0.296   0.184   0.043   0.773
B G  4U2XA                 38    0.197  15.520  -1.746   0.268   0.043   0.689
B M  4U2XA                 39    0.163  32.616  -0.121   0.453   0.248   0.299
B A  4U2XA                 40    0.182  20.045  -0.515   0.352   0.332   0.316
B L  4U2XA                 41    0.087  15.893   0.189   0.487   0.370   0.144
B L  4U2XA                 42    0.088  16.113  -0.292   0.487   0.370   0.144
B H  4U2XA                 43    0.232  42.255  -0.332   0.478   0.309   0.214
E R  4U2XA                 44    0.289  66.112   0.268   0.453   0.248   0.299
B L  4U2XA                 45    0.081  14.886   0.149   0.428   0.171   0.402
E K  4U2XA                 46    0.448  92.133   0.112   0.278   0.093   0.628
E T  4U2XA                 47    0.387  53.649  -1.071   0.184   0.043   0.773
E N  4U2XA                 48    0.511  74.825  -1.319   0.113   0.043   0.844
E D  4U2XA                 49    0.337  48.619  -1.254   0.113   0.087   0.800
B F  4U2XA                 50    0.162  32.574  -0.523   0.118   0.150   0.732
B A  4U2XA                 51    0.166  18.326   0.313   0.191   0.086   0.723
E P  4U2XA                 52    0.405  57.455  -0.568   0.354   0.048   0.598
E A  4U2XA                 53    0.404  44.565  -0.335   0.561   0.047   0.393
B W  4U2XA                 54    0.152  36.580  -0.242   0.660   0.049   0.291
E S  4U2XA                 55    0.420  49.259  -0.223   0.660   0.049   0.291
E M  4U2XA                 56    0.379  75.818  -0.340   0.622   0.015   0.363
B T  4U2XA                 57    0.212  29.418  -0.642   0.622   0.015   0.363
E R  4U2XA                 58    0.324  74.288   0.182   0.622   0.015   0.363
E N  4U2XA                 59    0.453  66.275  -0.914   0.622   0.015   0.363
B L  4U2XA                 60    0.096  17.578   0.108   0.622   0.015   0.363
B F  4U2XA                 61    0.069  13.868   0.253   0.622   0.015   0.363
E P  4U2XA                 62    0.356  50.531   0.147   0.717   0.014   0.269
E H  4U2XA                 63    0.350  63.665  -0.354   0.802   0.014   0.185
B L  4U2XA                 64    0.089  16.241  -0.385   0.802   0.014   0.185
B F  4U2XA                 65    0.137  27.496  -0.418   0.622   0.015   0.363
E Q  4U2XA                 66    0.620 110.750   0.577   0.339   0.016   0.645
E N  4U2XA                 67    0.337  49.395   0.015   0.053   0.005   0.942
E P  4U2XA                 68    0.426  60.492  -1.120   0.016   0.005   0.979
E N  4U2XA                 69    0.629  92.115  -0.653   0.053   0.005   0.942
E S  4U2XA                 70    0.416  48.814  -0.430   0.257   0.016   0.727
E T  4U2XA                 71    0.312  43.302   0.369   0.455   0.046   0.498
B I  4U2XA                 72    0.080  14.819  -0.146   0.561   0.047   0.393
E E  4U2XA                 73    0.291  50.750  -0.080   0.660   0.049   0.291
E S  4U2XA                 74    0.393  46.024  -0.351   0.622   0.015   0.363
B P  4U2XA                 75    0.105  14.885  -0.284   0.858   0.002   0.139
B L  4U2XA                 76    0.106  19.335  -0.077   0.923   0.002   0.076
B W  4U2XA                 77    0.140  33.790  -0.087   0.970   0.001   0.030
B A  4U2XA                 78    0.084   9.279  -1.381   0.975   0.003   0.022
B L  4U2XA                 79    0.049   8.935   0.502   0.975   0.003   0.022
B R  4U2XA                 80    0.197  45.067   0.725   0.975   0.003   0.022
B V  4U2XA                 81    0.091  14.033   0.264   0.975   0.003   0.022
B I  4U2XA                 82    0.075  13.912   0.412   0.938   0.007   0.055
B L  4U2XA                 83    0.072  13.202   0.017   0.938   0.007   0.055
B A  4U2XA                 84    0.163  17.974  -0.948   0.802   0.014   0.185
B A  4U2XA                 85    0.144  15.836  -0.359   0.660   0.049   0.291
B G  4U2XA                 86    0.213  16.739  -0.852   0.455   0.046   0.498
B I  4U2XA                 87    0.112  20.812   0.190   0.354   0.048   0.598
E Q  4U2XA                 88    0.476  85.049   0.585   0.455   0.046   0.498
E D  4U2XA                 89    0.432  62.280  -0.671   0.522   0.016   0.462
E Q  4U2XA                 90    0.491  87.764   0.423   0.717   0.014   0.269
E L  4U2XA                 91    0.373  68.388   0.118   0.802   0.014   0.185
B I  4U2XA                 92    0.140  25.808   0.425   0.802   0.014   0.185
E D  4U2XA                 93    0.416  59.874   0.007   0.802   0.014   0.185
E Q  4U2XA                 94    0.553  98.855   0.395   0.717   0.014   0.269
E S  4U2XA                 95    0.430  50.431  -0.381   0.622   0.015   0.363
B L  4U2XA                 96    0.153  27.978   0.155   0.522   0.016   0.462
B I  4U2XA                 97    0.124  22.940   0.261   0.522   0.016   0.462
E E  4U2XA                 98    0.536  93.674   0.650   0.600   0.003   0.397
B P  4U2XA                 99    0.233  32.992  -1.668   0.694   0.003   0.303
B L  4U2XA                100    0.084  15.435  -0.548   0.858   0.002   0.139
E A  4U2XA                101    0.301  33.203  -0.533   0.923   0.002   0.076
E G  4U2XA                102    0.317  24.948  -1.560   0.923   0.002   0.076
B A  4U2XA                103    0.060   6.590  -0.399   0.923   0.002   0.076
B L  4U2XA                104    0.077  14.062  -0.426   0.938   0.007   0.055
E G  4U2XA                105    0.297  23.382  -0.398   0.879   0.010   0.111
B L  4U2XA                106    0.115  21.057   0.239   0.879   0.010   0.111
B I  4U2XA                107    0.058  10.767   0.350   0.802   0.014   0.185
B S  4U2XA                108    0.191  22.373  -0.626   0.802   0.014   0.185
E D  4U2XA                109    0.341  49.080  -0.347   0.802   0.014   0.185
B W  4U2XA                110    0.095  22.751  -0.023   0.831   0.044   0.125
B L  4U2XA                111    0.083  15.216   0.227   0.751   0.050   0.199
B L  4U2XA                112    0.139  25.396   0.371   0.779   0.100   0.120
B T  4U2XA                113    0.182  25.188   0.022   0.701   0.107   0.192
E T  4U2XA                114    0.358  49.613  -0.245   0.502   0.102   0.396
B N  4U2XA                115    0.260  38.064  -1.007   0.307   0.165   0.527
B T  4U2XA                116    0.292  40.445  -0.488   0.191   0.086   0.723
E N  4U2XA                117    0.483  70.726  -1.225   0.118   0.150   0.732
B H  4U2XA                118    0.268  48.658  -0.801   0.066   0.296   0.638
B F  4U2XA                119    0.136  27.396  -0.079   0.074   0.484   0.442
E N  4U2XA                120    0.375  54.885  -0.631   0.074   0.484   0.442
B M  4U2XA                121    0.129  25.913  -0.096   0.069   0.386   0.545
E R  4U2XA                122    0.340  77.791   0.442   0.125   0.227   0.648
E T  4U2XA                123    0.320  44.342  -0.864   0.386   0.097   0.517
E Q  4U2XA                124    0.537  95.926  -0.578   0.502   0.102   0.396
E R  4U2XA                125    0.325  74.494   0.554   0.701   0.107   0.192
B V  4U2XA                126    0.103  15.800  -0.509   0.831   0.044   0.125
E K  4U2XA                127    0.330  67.922  -0.060   0.879   0.010   0.111
E E  4U2XA                128    0.411  71.749  -0.486   0.938   0.007   0.055
E Q  4U2XA                129    0.373  66.529   0.370   0.938   0.007   0.055
B L  4U2XA                130    0.073  13.348  -0.089   0.879   0.010   0.111
B S  4U2XA                131    0.169  19.854  -0.381   0.879   0.010   0.111
B L  4U2XA                132    0.243  44.457  -0.618   0.923   0.002   0.076
E K  4U2XA                133    0.425  87.525   0.413   0.923   0.002   0.076
B M  4U2XA                134    0.093  18.689  -0.236   0.970   0.001   0.030
B L  4U2XA                135    0.089  16.333   0.390   0.970   0.001   0.030
E S  4U2XA                136    0.384  45.005   0.839   0.970   0.001   0.030
B L  4U2XA                137    0.155  28.472   0.261   0.988   0.000   0.012
B I  4U2XA                138    0.066  12.154  -0.100   0.970   0.001   0.030
B R  4U2XA                139    0.231  53.013   0.107   0.970   0.001   0.030
E S  4U2XA                140    0.397  46.552  -0.758   0.970   0.001   0.030
B N  4U2XA                141    0.229  33.511  -0.640   0.923   0.002   0.076
B I  4U2XA                142    0.039   7.252  -0.286   0.970   0.001   0.030
E L  4U2XA                143    0.273  49.895   0.597   0.970   0.001   0.030
E K  4U2XA                144    0.501 103.035   0.968   0.923   0.002   0.076
B F  4U2XA                145    0.071  14.250  -0.126   0.923   0.002   0.076
B I  4U2XA                146    0.072  13.357  -0.379   0.879   0.010   0.111
E N  4U2XA                147    0.465  68.091   0.168   0.802   0.014   0.185
E K  4U2XA                148    0.399  82.115   0.638   0.717   0.014   0.269
B L  4U2XA                149    0.098  17.889  -0.057   0.522   0.016   0.462
B D  4U2XA                150    0.221  31.861  -0.446   0.386   0.097   0.517
B A  4U2XA                151    0.132  14.502  -0.548   0.231   0.330   0.439
B L  4U2XA                152    0.065  11.920   0.022   0.079   0.592   0.329
B H  4U2XA                153    0.146  26.576   0.447   0.085   0.775   0.140
B V  4U2XA                154    0.050   7.624   0.353   0.085   0.775   0.140
B V  4U2XA                155    0.058   8.899   0.553   0.085   0.775   0.140
B N  4U2XA                156    0.251  36.776  -0.189   0.074   0.484   0.442
B Y  4U2XA                157    0.316  67.422  -0.441   0.064   0.216   0.721
E N  4U2XA                158    0.558  81.735  -1.338   0.018   0.047   0.935
E G  4U2XA                159    0.445  35.006  -1.724   0.018   0.019   0.964
E L  4U2XA                160    0.393  71.903   0.062   0.019   0.141   0.840
B L  4U2XA                161    0.252  46.214  -1.065   0.005   0.336   0.660
E S  4U2XA                162    0.472  55.330  -1.110   0.004   0.420   0.576
E S  4U2XA                163    0.398  46.634  -0.390   0.003   0.718   0.279
B I  4U2XA                164    0.129  23.958  -0.155   0.002   0.816   0.182
E E  4U2XA                165    0.472  82.493   0.164   0.001   0.900   0.099
B I  4U2XA                166    0.164  30.414  -0.524   0.001   0.900   0.099
E G  4U2XA                167    0.366  28.828  -0.745   0.003   0.718   0.279
B T  4U2XA                168    0.234  32.400  -1.087   0.004   0.514   0.481
E Q  4U2XA                169    0.534  95.319  -0.285   0.005   0.336   0.660
E N  4U2XA                170    0.610  89.363  -1.336   0.004   0.138   0.858
B H  4U2XA                171    0.261  47.476   0.356   0.004   0.514   0.481
E T  4U2XA                172    0.296  41.097   0.363   0.001   0.900   0.099
B I  4U2XA                173    0.052   9.712   1.017   0.001   0.959   0.040
B I  4U2XA                174    0.085  15.781   0.540   0.001   0.959   0.040
B I  4U2XA                175    0.047   8.714   0.541   0.001   0.959   0.040
B T  4U2XA                176    0.124  17.227  -0.045   0.001   0.959   0.040
B R  4U2XA                177    0.221  50.517   0.138   0.002   0.816   0.182
B T  4U2XA                178    0.173  24.023  -0.483   0.004   0.514   0.481
E N  4U2XA                179    0.390  57.155  -0.942   0.021   0.279   0.699
B M  4U2XA                180    0.143  28.674  -0.346   0.021   0.279   0.699
B G  4U2XA                181    0.099   7.799  -1.830   0.023   0.655   0.322
B F  4U2XA                182    0.104  20.853   0.119   0.018   0.846   0.136
B L  4U2XA                183    0.094  17.230   0.165   0.011   0.918   0.071
B V  4U2XA                184    0.081  12.434   0.121   0.011   0.918   0.071
E E  4U2XA                185    0.315  55.048   0.353   0.011   0.918   0.071
B L  4U2XA                186    0.095  17.303   0.189   0.018   0.846   0.136
E Q  4U2XA                187    0.436  77.941   0.513   0.021   0.756   0.223
E E  4U2XA                188    0.436  76.152   0.410   0.021   0.451   0.528
E P  4U2XA                189    0.259  36.795  -0.341   0.052   0.084   0.864
E D  4U2XA                190    0.523  75.350  -0.257   0.181   0.016   0.803
E K  4U2XA                191    0.593 121.939   0.158   0.522   0.016   0.462
E S  4U2XA                192    0.548  64.179  -0.499   0.622   0.015   0.363
B A  4U2XA                193    0.262  28.894  -1.019   0.622   0.015   0.363
E M  4U2XA                194    0.326  65.333  -0.944   0.622   0.015   0.363
E N  4U2XA                195    0.567  83.082  -0.652   0.522   0.016   0.462
E R  4U2XA                196    0.522 119.584  -0.528   0.354   0.048   0.598
E M  4U2XA                197    0.484  96.888  -0.583   0.113   0.043   0.844
E K  4U2XA                198    0.558 114.678  -0.183   0.053   0.043   0.903
E P  4U2XA                199    0.540  76.654  -1.466   0.018   0.019   0.964
E G  4U2XA                200    0.432  34.014  -1.526   0.018   0.019   0.964
E P  4U2XA                201    0.419  59.442  -0.976   0.053   0.043   0.903
B A  4U2XA                202    0.288  31.727  -1.172   0.056   0.142   0.802
E K  4U2XA                203    0.489 100.505   0.634   0.069   0.386   0.545
B F  4U2XA                204    0.127  25.569   0.236   0.074   0.484   0.442
E S  4U2XA                205    0.359  42.122  -0.217   0.169   0.612   0.219
B L  4U2XA                206    0.152  27.813   0.021   0.159   0.516   0.325
B L  4U2XA                207    0.152  27.795  -0.055   0.231   0.330   0.439
B H  4U2XA                208    0.239  43.401   0.081   0.453   0.248   0.299
E E  4U2XA                209    0.531  92.748   0.287   0.605   0.105   0.290
E S  4U2XA                210    0.465  54.557  -0.847   0.561   0.047   0.393
B T  4U2XA                211    0.308  42.664  -0.265   0.561   0.047   0.393
B L  4U2XA                212    0.215  39.330  -0.166   0.561   0.047   0.393
E K  4U2XA                213    0.619 127.411   0.881   0.502   0.102   0.396
E A  4U2XA                214    0.508  55.971  -0.587   0.455   0.046   0.498
E F  4U2XA                215    0.269  54.089  -0.642   0.268   0.043   0.689
E T  4U2XA                216    0.826 114.622  -0.006   0.003   0.003   0.994

Explain the output.  Go back.
```

---
